# Network pharmacology-based strategy to investigate pharmacological mechanisms of Zuojinwan for treatment of gastritis

**DOI:** 10.1186/s12906-018-2356-9

**Published:** 2018-11-01

**Authors:** Guohua Yu, Wubin Wang, Xu Wang, Meng Xu, Lili Zhang, Lei Ding, Rui Guo, Yuanyuan Shi

**Affiliations:** 1 School of Life Sciences, Beijing University of Chinese Medicine, No.11 East road, North 3rd Ring Road, Beijing, 100029 China; 2 Shenzhen Hospital, Beijing University of Chinese Medicine, No. 1 Dayun road, Sports New City Road, Shenzhen, 518172 China

**Keywords:** Zuojinwan, Network pharmacology, Gastritis

## Abstract

**Background:**

Zuojinwan (ZJW), a classic herbal formula, has been extensively used to treat gastric symptoms in clinical practice in China for centuries. However, the pharmacological mechanisms of ZJW still remain vague to date.

**Methods:**

In the present work, a network pharmacology-based strategy was proposed to elucidate its underlying multi-component, multi-target, and multi-pathway mode of action against gastritis. First we collected putative targets of ZJW based on TCMSP and STITCH databases, and a network containing the interactions between the putative targets of ZJW and known therapeutic targets of gastritis was built. Then four topological parameters, “degree”, “betweenness”, “closeness”, and “coreness” were calculated to identify the major targets in the network. Furthermore, the major hubs were imported to the Metacore database to perform a pathway enrichment analysis.

**Results:**

A total of 118 nodes including 59 putative targets of ZJW were picked out as major hubs in terms of their topological importance. The results of pathway enrichment analysis indicated that putative targets of ZJW mostly participated in various pathways associated with anti-inflammation response, growth and development promotion and G-protein-coupled receptor signaling. More importantly, five putative targets of ZJW (EGFR, IL-6, IL-1β, TNF-α and MCP-1) and two known therapeutic targets of gastritis (CCKBR and IL-12β) and a link target NF-κB were recognized as active factors involved in the main biological functions of treatment, implying the underlying mechanisms of ZJW acting on gastritis.

**Conclusion:**

ZJW could alleviate gastritis through the molecular mechanisms predicted by network pharmacology, and this research demonstrates that the network pharmacology approach can be an effective tool to reveal the mechanisms of traditional Chinese medicine (TCM) from a holistic perspective.

**Electronic supplementary material:**

The online version of this article (10.1186/s12906-018-2356-9) contains supplementary material, which is available to authorized users.

## Background

Gastritis is an acute or chronic, diffuse or focal inflation of the lining of the stomach [1]. It is brought on by many factors, including infection by *Helicobacter pylori*, drug induced such as aspirin, Non-Steroidal Anti-Inflammatory Drugs (NSAIDs), corticosteroids and alcohol consumption [2]. The most frequent symptoms of gastritis include upper abdominal pain, heartburn, nausea, and vomiting [3, 4]. Gastritis is believed to affect about half of people in the world and it generates considerable costs to society [5]. Although current therapies, including antacids, H-2 blockers, proton pump inhibitors and antibiotics can alleviate some major symptoms of gastritis, these medications have also triggered a series of serious side effects like abdominal pain (or stomach pain), constipation, and diarrhea. Therefore, it is still necessary to find novel and safe prevention strategies.

Traditional Chinese medicine (TCM) is a comprehensive medicinal system that plays an important role in health maintenance for Asian people, and has gradually gained popularity in western countries due to the reliable therapeutic efficacy and fewer side effects [6, 7]. Based on the theory of traditional Chinese herbal medical science, TCM offers bright prospects for the prevention and treatment of complex diseases such as gastritis in a systematic way [8].

Zuojinwan, which consists of *R. coptidis* and *E. rutaecarpa* powder in the ratio of 6: 1(*w*/w), is a famous Chinese medicine prescription used for treatment of gastric diseases [9, 10]. ZJW was first recorded in a famous ancient medicine treatise *Danxi Xinfa* in Yuan Dynasty of Chinese history (1271 AD–1368 AD), and has been approved by the China Food and Drug Administration (CFDA). From the bench to the bedside, previous researches on ZJW for gastric diseases could be mainly documented into two sections: (1) Clinical practices show that ZJW has been widely used for treating gastric diseases. (2) Basic researches indicate that ZJW exerts a range of pharmacological activities, including anti-inflammation, anti-ulcer and anti-acid activities and inhibitory effect on the growth of *Heliobacter pylori*. Although well-practiced in clinical medicine, very little is known about the active substances and specific molecular mechanisms of ZJW acting on gastritis.

Similar to other TCM formulas, ZJW is a multi-component and multi-target agent that achieves its specific therapeutic efficacy through active components that regulate molecular networks within the body. Therefore, it is hard to investigate the pharmacological mechanisms of ZJW in the treatment of gastritis. With the rapid progress of bioinformatics, systems biology and polypharmacology, network pharmacology-based approaches have been proven to be a powerful way for compatible and mechanistic exploration of TCM formula [7, 11, 12]. For example, Yu et al. used a network pharmacology method to analyze the synergistic mechanism of Yin-Huang-Qing-Fei capsule acting on chronic bronchitis [13]. Yue et al. developed an integrated system pharmacology approach, combined a number of network-based computational methods and algorithm-based approaches to clarify the mechanisms of Danggui-Honghua for treatment of blood stasis syndrome [14].

**Fig. 1:**
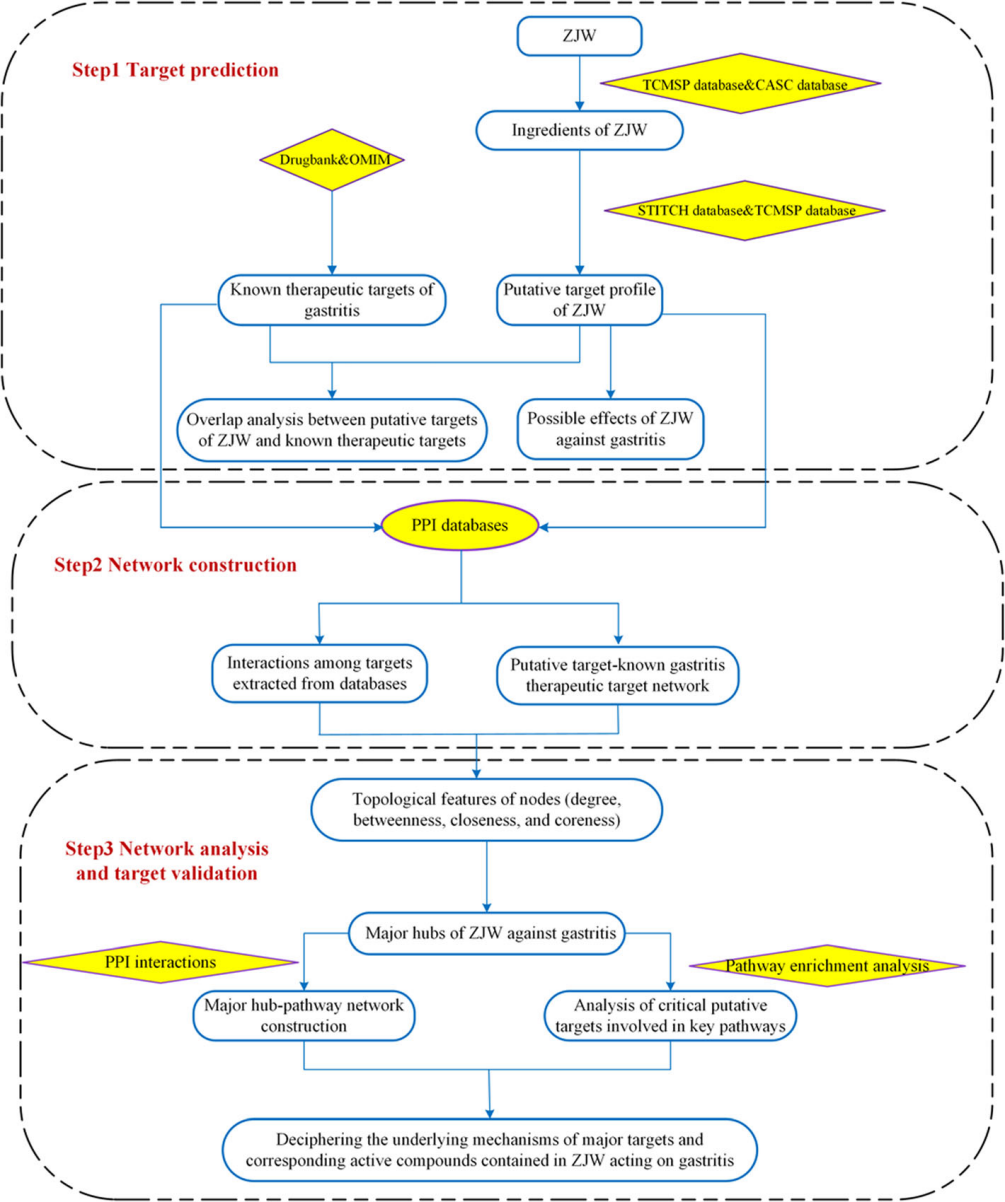
The flowchart of network pharmacology-based strategy for deciphering the mechanisms of ZJW acting on gastritis. Abbreviations: ZJW, Zuojinwan; TCMSP, Traditional Chinese Medicine Systems Pharmacology; CASC, Chinese Academy of Sciences Chemistry; OMIM, Online Mendelian Inheritance in Man; PPI, protein–protein interaction

In this work, we aim to use a comprehensive network pharmacology-based approach to investigate the mechanisms of how ZJW exerts the therapeutic effects on gastritis. The flowchart of the experimental procedures of our study was shown in Fig. 1.

## Methods

### Data preparation

#### Chemical ingredients database building

To determine the chemical ingredients of the two herbs contained in ZJW, we performed a search by Traditional Chinese Medicine Systems Pharmacology Database [15] (TCMSP, http://lsp.nwu.edu.cn/tcmsp.php, updated on May 31, 2014) and Chinese Academy of Sciences Chemistry Database [16] (CASC, http://www.organchem.csdb.cn/scdb/main/slogin.asp, updated on February 11, 2018) and related literatures using “*R. coptidis*” and “*E. rutaecarpa*” as the queries. TCMSP is a unique systems pharmacology database of Chinese herbal medicines which captures the herbs, chemicals, targets and drug-target networks [17]. CASC, one of the most comprehensive chemical databases in the world, can provide the chemical information of traditional Chinese herbs and natural products [13].

#### The prediction of known therapeutic targets acting on gastritis

We collected gastritis targets from two sources. One was the DrugBank database [18] (http://www.drugbank.ca/, version 4.3), which is a unique bioinformatics and cheminformatics resource that combines detailed drug data with comprehensive drug target information. The keyword “gastritis” was used and only drug–target interactions for drugs approved by the Food and Drug Administration (FDA) for treating gastritis and human gene/protein targets were selected. The other resource was the Online Mendelian Inheritance in Man (OMIM) database [19] (http://www.omim.org/, updated on May 4, 2018). The OMIM database catalogued all known diseases with a genetic component and linked them to the relevant genes in the human genome and provided references for further research and tools for genomic analysis of a catalogued gene [19]. We searched the OMIM database with the query “gastritis” as well.

#### The prediction of putative targets of the ingredients within ZJW

The integrative efficacy of the ingredients in ZJW was determined by analyzing the ingredients and targets interactions obtained from TCMSP Database and STITCH DataBase [20] (http://stitch.embl.de/, ver. 4.0) with the species limited as “*Homo sapiens*”. STITCH was a database to explore known and predicted interactions between chemicals and proteins. Only the proteins which had direct interactions with each chemical in ZJW were selected as the putative targets.

#### Protein–protein interaction data

Protein–protein interaction (PPI) data were derived from eight major existing public PPI databases, namely, String [21], Reactome [22], Online Predicted Human Interaction Database (OPHID) [23], InAct [24], Human Protein Reference Database (HPRD) [25], Molecular Interaction Database (MINT) [26], Database of Interacting Proteins (DIP) [27], and PDZBase [28]. The eight open databases covered the majority of known human protein-protein interactions information. Detailed information about these PPI databases was provided in Additional file 1: Table S1. All the data were merged after removing redundant entries and the differing ID types of the proteins were converted to UniProt IDs.

### Network construction

The putative targets of ZJW, the gastritis disease targets and interactional proteins were connected based on the protein-protein interactions derived from the eight public databases. Then the interactions between proteins of the putative targets of ZJW, known therapeutic targets for gastritis and interactional human targets were combined to construct putative ZJW target-known therapeutic targets of gastritis network. It can be applied to illustrate the relationships between putative targets contained in ZJW and known therapeutic targets of gastritis. The graphical interactions in this network was visualized using Cytoscape software [29] (version 3.6.0, Boston, MA, USA). In addition, a null model was constructed to verify the significance of the putative ZJW target-known therapeutic targets of gastritis network.

#### Putative ZJW target-known therapeutic targets of the gastritis network

Based on the interactions between putative ZJW targets, known therapeutic targets of gastritis and interactional human proteins, the whole links of the network were established. According to previous reports [30–32], a node would be defined as a hub when the degree of the node was more than twofold the median degree of all the nodes in the same network. After selecting hubs from the network, the direct interactions among hubs was constructed. Next, we used four topological parameters, “degree”, “betweenness”, “closeness”, and “coreness” to evaluate the topological importance of the selected hubs. The definitions for each topological parameter mentioned above are given in ‘Definitions of topological propertie’ section. Only the hubs whose topological parameters were greater than the corresponding median values were considered as major hubs.

#### Definitions of topological properties

As for each node i in the networks, the definitions of four measures for evaluating its topological properties were shown as follows. ‘Degree’ is defined as the number of links to node i. ‘Node betweenness’ represents the number of the shortest paths between pairs of nodes that ran through node i. The concept of ‘Node closeness’ is the inverse of the sum of distances from the node i to all other nodes. The closeness centrality can also be seen as a metric of the time it will take to sequentially spread information from node i to all the other accessible nodes. We usually use ‘Degree’, ‘Node betweenness’ and ‘Node closeness centralities’ to describe a protein’s topological importance in the network. In other words, the relationship between these three topological parameters and importance of corresponding protein is direct proportional [33]. ‘Coreness’ is shell index of ‘K-core’ decomposition. K-core analysis is an iterative procedure in which the nodes are removed from the networks in descending degree order. The highest degree node was selected as the main core or the highest k-core of the network. After repeatedly deleting vertices from the network whose degree is less than k, a k-core sub-network of the original network forms. This process generates a series of sub-networks that uncover the main hierarchical layers of the original network step by step. On this basis, ‘Coreness’ is a parameter to measure the centrality of node i.

#### Null model construction

In order to validate the non-trivial structure of putative ZJW target-known therapeutic targets of the gastritis network, we constructed a null model in which all the nodes of the ZJW network were conserved as well as the number of links, but the nodes were rewired randomly in terms of the Erdös-Rényi model. We also used the corresponding topological parameters to screen out the major hubs in the null model.

### Pathway enrichment performance

To cluster the biological functions of the major hubs, they were uploaded to MetaCore™ [34] (https://portal.genego.com) as Gene Symbol style to run pathway enrichment analysis. MetaCore online database delivers high-quality biological systems content as well as essential data and analytics, including sophisticated integrated pathway and network analysis for multi-omics data. First the list of major hubs was input into the Metacore database as a new event, we selected the new event in the online database and clicked on the icons with “protein functional annotation and enrichment analysis” and “network construction and analysis” successively to obtain the main pathways and network distribution involved in the functional regulation. Then the results could be analyzed in the next step. Also, we calculated and evaluated significant pathways assisted by Database Visualization and Integrated Discovery system [35] (DAVID, http://david.abcc.ncifcrf.gov/home.jsp, version 6.7) and the Kyoto Encyclopedia of Genes and Genomes database [36] (KEGG, http://www.genome.jp/kegg/, updated on April 18, 2016). Then we compared the generated results with a null model to assess the statistical significance.

## Results

### Composite ingredients of ZJW

A total of 170 chemical ingredients (Additional file 2: Table S2) of the two herbal medicines in ZJW were retrieved from TCMSP and CASC and related literatures, including 29 ingredients in *R. coptidis* and 141 ingredients in *E. rutaecarpa*.

### Known therapeutic targets acting on gastritis

In total, 75 known therapeutic targets for gastritis were collected from DrugBank database. And 15 known therapeutic targets for the treatment of gastritis were acquired based on OMIM database. After eliminating the redundancy, a total of 90 known therapeutic targets in the treatment of gastritis were collected in this study. The details were described in Additional file 3: Table S3.

### Putative targets for ZJW

According to the target prediction system in TCMSP and STITCH databases, the quantity of putative targets for *R. coptidis* and *E. rutaecarpa* was 175 and 348, respectively. There were 106 putative targets of the two herbs overlapped, which was suggestive of potential interactions between *R. coptidis* and *E. rutaecarpa* in the course of treatment. Detailed information about putative targets is provided in Additional file 4: Table S4.

### Network and pathway analysis

To shed light on the potential mechanisms of ZJW acting on gastritis, the putative ZJW target-gastritis related target network consisting of putative ZJW targets, known therapeutic target for gastritis and interactional human proteins was constructed based on PPI databases. As a result, the network was composed of 5559 nodes and 21567 edges. For detailed information about this network, see Additional file 5: Table S5.

A hub target in a network is regarded as a crucial node and used to measure the essence of the whole network. It has been reported that [37], nodes will be defined as hubs if their degree is greater than twofold the median degree of all nodes in the network. In this network, we used the twofold median value of node degree (two) as a cutoff point primarily. After removing the targets whose degree was less than or equal to two, 2654 nodes were encoded as hubs. Then four topological features, “degree”, “betweenness”, “closeness”, and “coreness”, of selected hubs were calculated to screen the major hubs in the network. Only the hubs with higher values of “degree”, “betweenness”, “closeness”, and “coreness” (above the median value of all the network nodes) were identified as the major hub targets. To focus on the further enrichment analysis of crucial hubs, we discarded some targets which might play unimportant roles in the network according to the topological features. Therefore, we kept proteins with “degree” > 4, “betweenness” > 0.0002, “closeness” > 0.3919 and “coreness” > 5 as major hubs. Eventually, 118 major hubs were picked out for further study, of which 59 targets were from putative targets of ZJW and 12 targets were derived from known therapeutic target of gastritis. The details were shown in Additional file 6: Table S6.

**Fig. 2:**
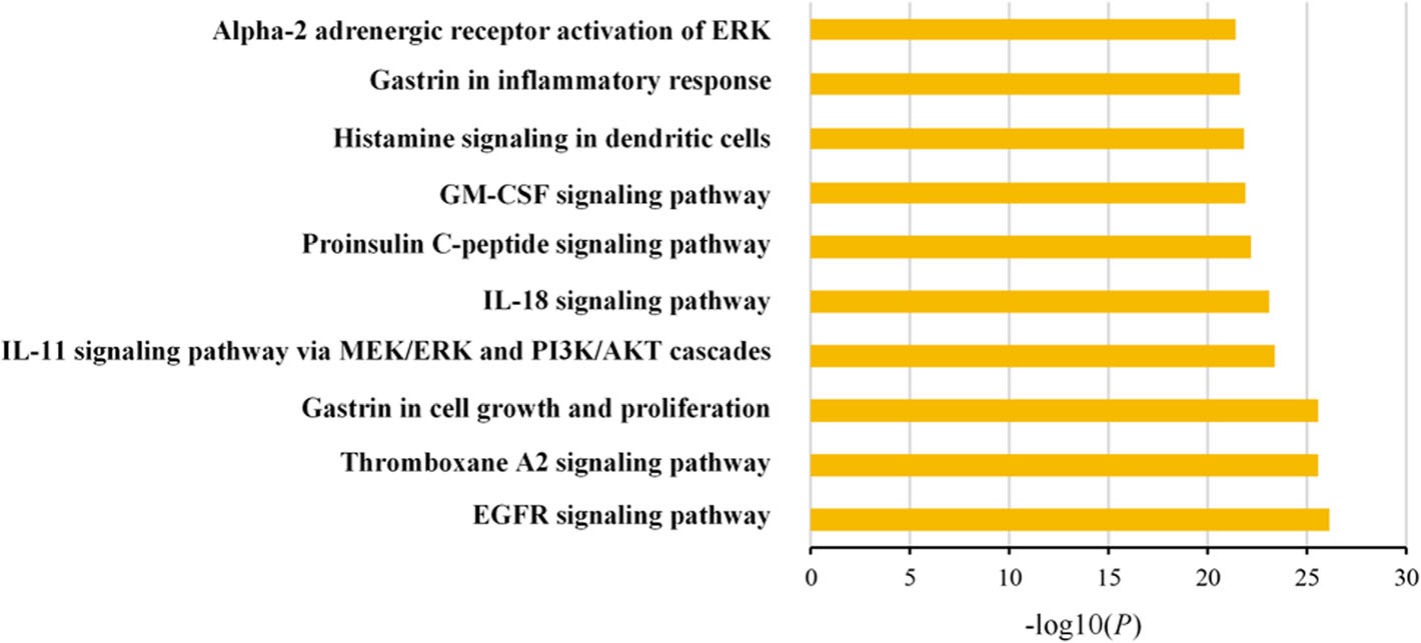
Main pathways enriched by major hubs from Metacore database. The top 10 pathways measured by counts were selected to demonstrate the crucial biological actions of major hubs. The abscissa stands for target counts in each pathway; and the ordinate stands for main pathways

To clarify the biological actions of these hubs, a pathway enrichment analysis was performed based on Metacore database. We fed MetaCore a gene list of major hubs, generating relevant pathways which might have an important influence on the biological process for ZJW treating gastritis. Only pathways with *P*-value<0.05 were considered as significant pathways. The full list of significant pathways was shown in Additional file 7: Table S7. We analyzed the data and relevant biological processes, choosing top ten remarkable significant pathways according to the *P* value for further study. The top ten main pathways were shown in Fig. 2.

**Fig. 3:**
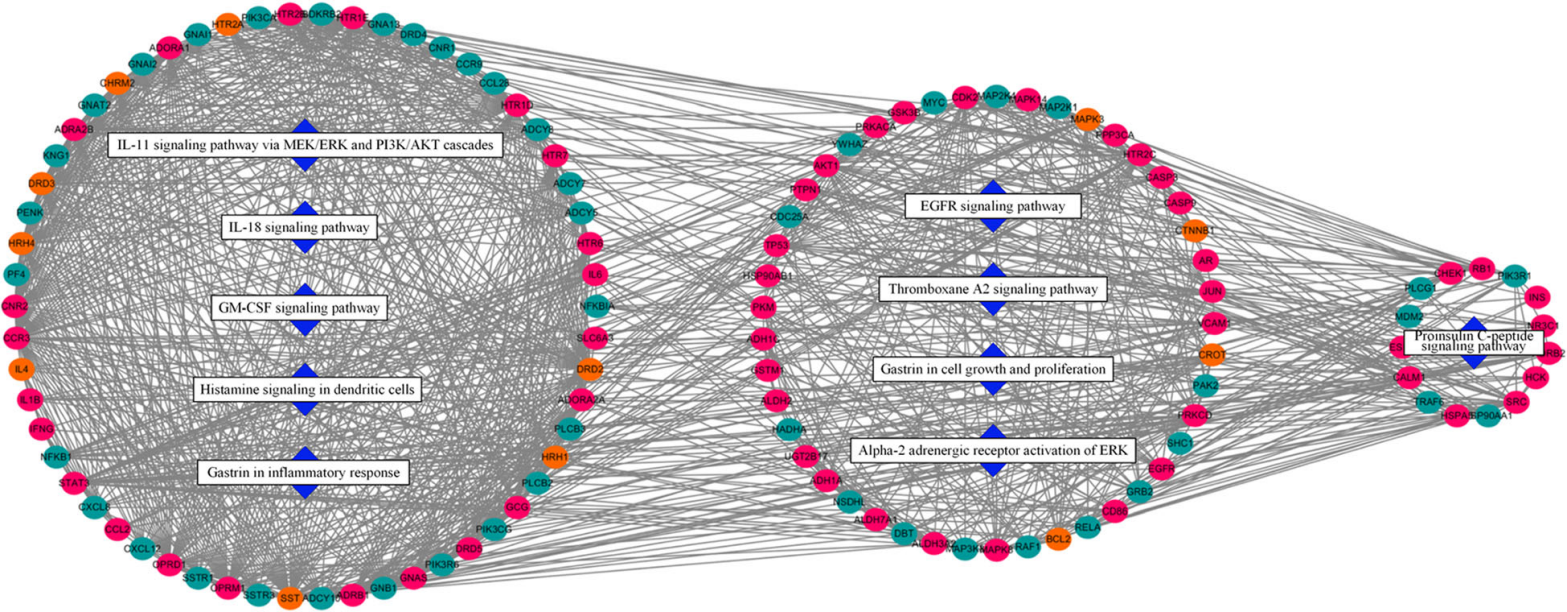
The interaction network between major hubs and main pathways. Round red nodes stand for putative targets of ingredients contained in ZJW; round orange nodes stand for known therapeutic targets for gastritis; round green nodes stand for the main link targets between red nodes and orange nodes; blue diamonds stand for main pathways based on enrichment analysis of major hubs

Modularity could be an important aspect of a network. Nodes highly interconnected within a network were usually participated in the same biological modules. In terms of the functional distribution of major hubs and main pathways, the interaction network of putative ZJW targets and gastritis-related targets and interactional targets was divided into three modules. The assortments of the main pathways and modules are shown in Fig. 3. The maximum module consisting of major hubs was associated with inflammation suppression and immune responses, and the second module was sorted as a progression of growth and development, while the minimum module was concentrated in G-protein-coupled receptor signaling.

**Fig. 4:**
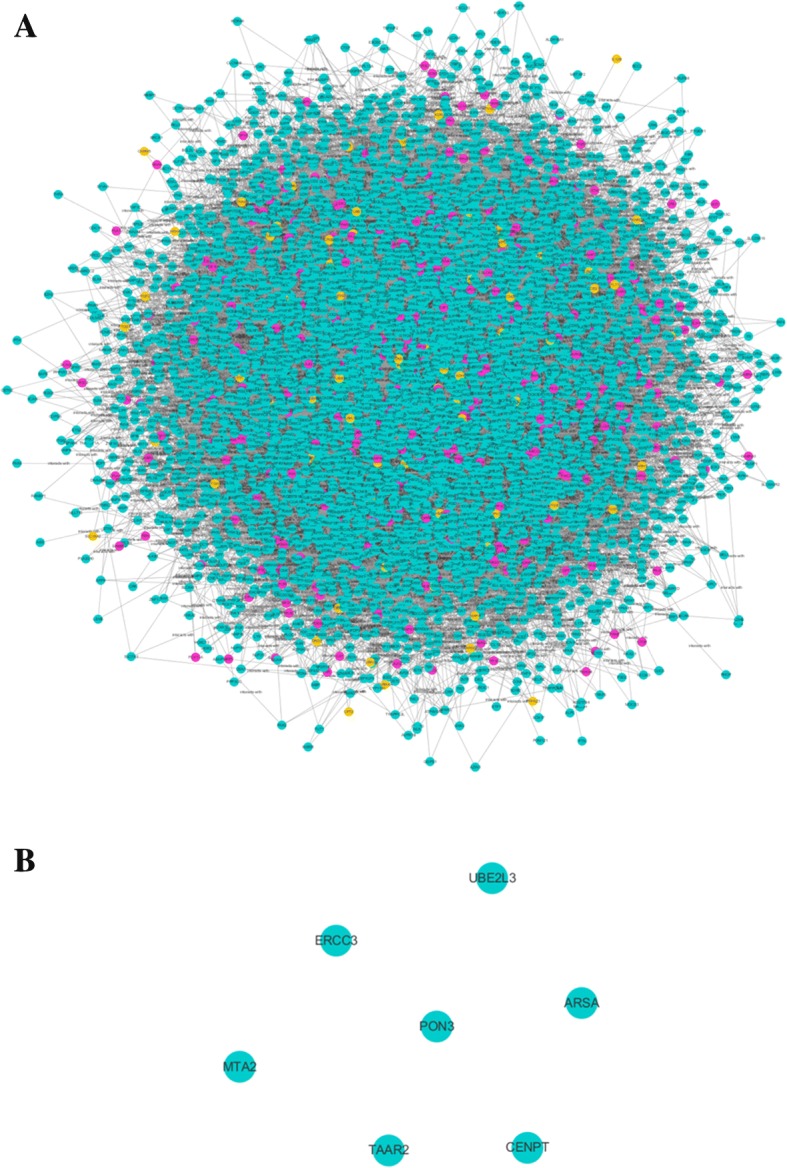
Null model network. **a** The null model network composed of the same nodes and the number of edges from ZJW network but randomized links based on Erdös-Renyí model. **b** The core structure of null model network after calculation of four topological properties

We analyzed the null-model-network topology and performed pathway enrichment analysis in the same way as we interpreted the ZJW network, and no modules or pathways were found since just a few genes without any interaction were screened out. The details of the whole null-model-network were shown in Additional file 8: Table S8. The major hubs of null-model- network were also supplemented in Additional file 9: Table S9. The null-model-network topology (Fig. 4a) and the core structure of the random network after calculation of four topological properties (Fig. 4b) were shown in Fig. 4. By contrast with the null model, the significance of ZJW network was verified.

### Potential mechanisms of ZJW in the treatment of gastritis

In the maximum module, cytokines and genes were enriched in inflammatory pathways including IL-11 signaling pathway via MEK/ERK and PI3K/AKT cascades, IL-18 signaling pathway, GM-CSF signaling pathway, histamine signaling in dendritic cells and gastrin in inflammatory response. Most of these pathways contain PI3K/AKT signaling pathway and NF-κB mediated signaling pathway, and both play important roles in inflammatory response as well as cell proliferation. Usually, the extracellular cytokine binding to its corresponding receptor leads to a series of activation of downstream molecules, and sequentially PI3K/AKT signaling pathway or NF-κB mediated signaling pathway can be activated, resulting in transcription of relevant transcription factors and secretion of some inflammatory factors participating in the process of inflammation.

EGFR signaling pathway occupied a leading position in the second module. The epidermal growth factor receptor (EGFR) is a cell surface protein that binds to epidermal growth factor. Binding of the protein to an extracellular growth factor ligand induces receptor dimerization and tyrosine autophosphorylation and leads to diverse biologic responses, including cell proliferation, differentiation, cell motility and survival [38]. EGFR dimerization and tyrosine autophosphorylation can activate several intracellular signaling pathways, such as PI3K/AKT signaling pathway, JUK signaling pathway. These pathways play an important role in cell growth and proliferation. As well, EGFR signaling pathway is partly involved in those main growth and development-related pathways including thromboxane A2 signaling pathway, gastrin in cell growth and proliferation, alpha-2 adrenergic receptor activation of ERK.

Proinsulin C-peptide signaling pathway composed a part of the minimum module. One of the putative receptor for proinsulin C-peptide was supposed to be a specific GPCR linked to the G-protein alpha-i family [39–41], which could stimulate PI3K/AKT and NF-κB. And then transcription of inflammatory genes and anti-apoptotic genes was activated by proinsulin C-peptide-stimulated NF-κB. A series of biological functions of inflammatory response and immune actions would be initiated. Furthermore, proinsulin C-peptide also participated in regulation ERK. And the binding of proinsulin C-peptide and GPCR triggered ERK signaling via PLC-beta-dependent stimulation of PKC or PI3K-dependent pathway [42]. These biological reactions contributed a lot to cell proliferation.

## Discussion

Gastritis can be caused by various factors, and some remarkable alterations of the stomach mucosa including epithelial damage and mucosal inflammation will appear once patients suffer from gastritis [43]. NF-κB mediated signaling pathway is activated while gastritis occurs. NF-κB can promote the expression of pro-inflammatory cytokines genes such as IL-1β, TNF-α, IL-12β, IL-6, IL-8 [44–49]. These cytokines are attributed to the inflammation of gastric epithelial cells. It has been reported that the injury of gastric mucosa barrier has a close relationship with gastritis, and long-playing activation by inflammatory factors will induce the severe lesion in gastric glandular tissues when the gastric mucosa is not well regenerated [50]. EGF is one of the most important factors in gastric tissue repair and cell regeneration, showing its effects through the combination with its receptor (EGFR) located in gastric epithelial basal or bilateral membrane. The level of the expression of EGFR can be highly detected in the damaged gastric barrier and perfused epithelia while it is rarely found in normal epithelial cells. It is also found that EGFR expressed more highly in the surface mucosal layer than the deep and muscle layers of stomach, implying its effects on the promotion of the growth and recovery of damaged gastric epithelial cells [51]. However, excessive expression of EGFR can induce malignant growth in gastric epithelial cells and may cause canceration to some extent [52, 53]. Therefore, to regulate the balance of the expression of EGFR is very pivotal in gastric mucosa development and repairment. Besides, as a multifunctional gastro-intestinal hormone, gastrin is secreted by G cells in the gastric antrum and plays important roles in stimulating acid secretion, cell growth and mucous regeneration [54, 55]. As a result of increase of serum concentration of gastrin accompanying with increased grades of atrophic gastritis, the serum gastrin level is taken as a significant biomarker for evaluating the status of gastric inflammation [56, 57]. It is also evident that gastrin is a growth factor which can induce gastric carcinogenesis [58, 59].

**Fig. 5:**
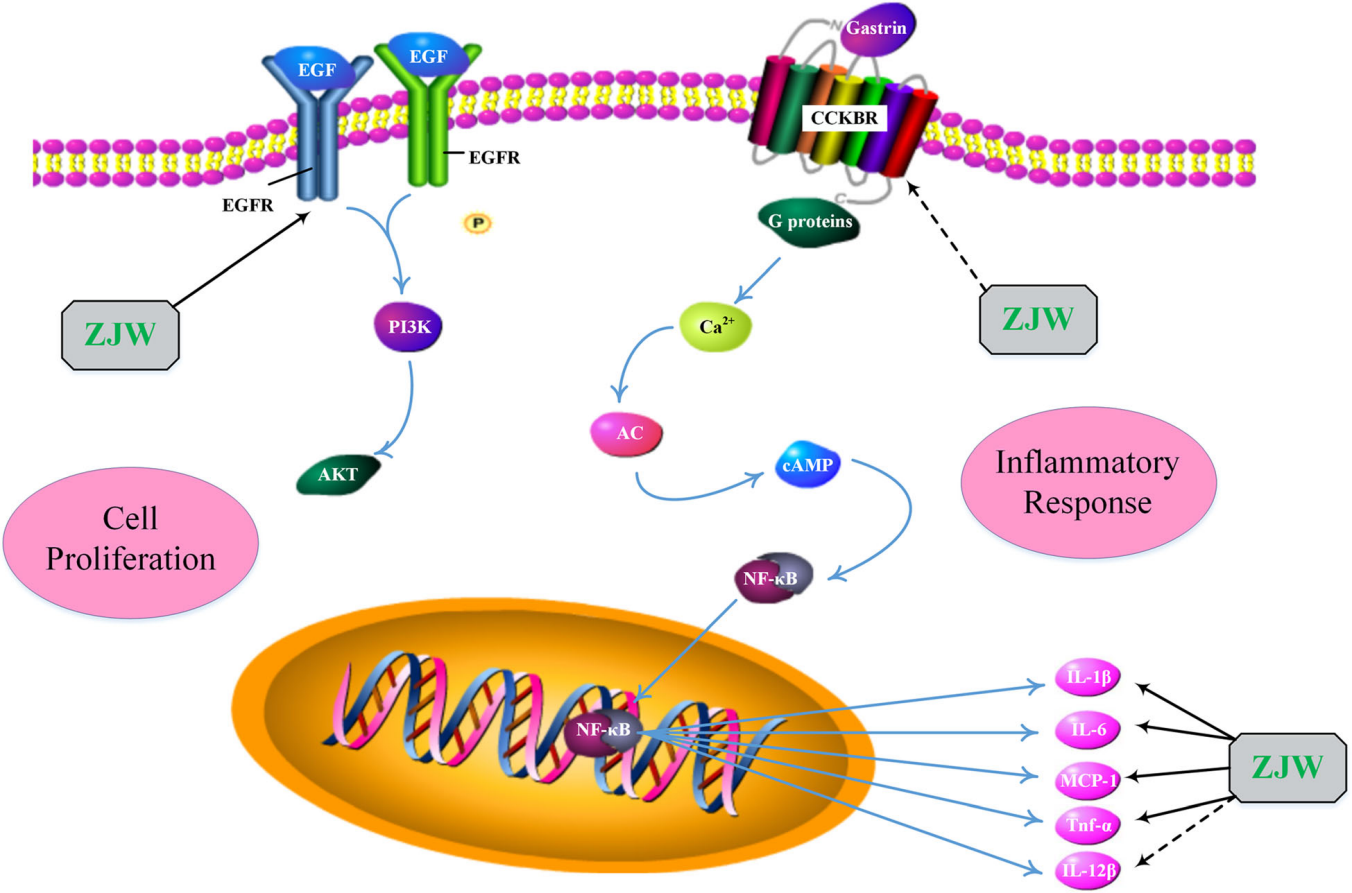
Illustration of crucial biological progress caused by putative targets and known therapeutic targets for gastritis. Abbreviations: EGF, epidermal growth factor; EGFR, epidermal growth factor receptor; CCKBR, cholecystokinin-B receptors; PI3K, phosphoinositide 3-kinase; AKT, protein kinase B; Ca^**2**+^, Calcium ion; AC, adenylyl cyclase; cAMP, cyclic adenosine monophosphate; NF-κB, nuclear factor kappa B; IL-1β, interleukin 1 beta; IL-6, interleukin 6; MCP-1, monocyte chemotactic protein-1; TNF-α, tumor necrosis factor alpha; IL-12β, interleukin 12 beta; ZJW, Zuojinwan

The pathological processes of gastritis are closely related to inflammatory response and cell development. Five putative targets of ZJW (interleukin-6[IL-6], IL-1β, tumor necrosis factor [TNF]-α, monocyte chemotactic protein [MCP]-1 and EGFR), and two known therapeutic targets of gastritis (cholecystokinin-B receptors[CCKBR] and IL-12β) and a link target between ZJW and disease NF-κB are shown to be the most active factors that participate in these key pathways, which are implied an important role in the occurrence and promotion of gastritis. The main biological processes are shown in Fig. 5.

Cytokines and chemokines can be produced when a signaling cascade is activated during the progression of lesion in the stomach, inducing chronic inflammatory response. Gastric epithelium can secret a series of chemokines including interleukin (IL)-6 and IL-1β, which are chemotactic for neutrophils and mononuclear cells. With the recruitment of neutrophils and macrophages in the mucosa of stomach, the infiltration is the main factor of a chronic gastritis. Expect neutrophils and mononuclear cells, dendritic cells and T and B cells can also form infiltration and then activate the production of monocyte chemotactic protein (MCP)-1, tumor necrosis factor (TNF)-α, IL-12 and so on [60]. IL-6 can be produced by T cells and macrophages that gives rise to immune response during infection or after injury which induces inflammation in the gastric mucosa or other tissues [61]. It is also a pleiotropic cytokine associated with the growth and differentiation of immunocyte as well as the expression of some other cytokines [62]. Furthermore, IL-6 has been reported as a significant marker in the process of inflammation-related canceration [63, 64]. And there is evidence that in an inflammation-induced tumor model, IL-6 deficiency could relive the promotion of tumor development [65, 66]. MCP-1, known as CC-chemokine ligand 2 (CCL2), is a micromolecular potent chemoattractant for leukocytes to gather on the position where tissue is injured and inflammation or tumor are triggered [60, 67]. Studies have demonstrated that many tumor cells can express CCL2, suggesting its role in the course of cancer [68–71]. It is also reported that tumor samples from 68 gastric cancer patients highly expressed CCL2, implied that CCL2 may have a close relationship with gastric disease. Thus, CCL2 is considered to be a predictive molecule for gastric carcinoma [72]. Secreted by monocytes and macrophages, TNF-α stimulates the production of a series of cytokine and play an important part in gastritis. TNF-α is suggested to be a considerable immune mediator in inflammatory response which is initiated by infection and other factors [73, 74]. It has a positive influence on gastric mucosal apoptosis, and persistent apoptosis may cause extensive gastric mucosa damage like gastric ulcer [75, 76]. IL-1β is referred to be a pro-inflammatory cytokine that can be largely produced as a result of host defense against external invasion and tissue lesions [77]. It is found that high expression of IL-1β in the stomach of transgenic mice can activate spontaneous severe gastric inflammatory response [78]. Therefore, IL-1β is an important signaling mediator in the promotion of gastritis. It is also emphasized on the investigation that the polymorphisms of IL1B gene may be involved in the high risk of the generation of gastric cancer [79–81]. Moreover, it is demonstrated that in a IL-1β-deficient mice model, the infiltration of immune cells and gastric tumors are positively suppressed [82]. Gastrin is identified as the chief stimulant of gastric acid secretion which is produced by G cells in the gastric antrum [50]. Gastrin exerts its effects primarily through binding to cholecystokinin-B receptors (CCKBR) on enterochromaffin-like (ECL) cells of the gastric mucosa [83, 84]. CCKBR, also known as cholecystokinin-2 receptors (CCK2R), is a seven transmembrane G-protein coupled receptor that is mostly expressed in gastric fundus [85]. The expression of gastrin-CCKBR indicates the atrophic changes in the stomach [54, 56]. Consequently, gastrin-CCKBR level is a vital standard for measuring the severity of gastritis [57]. In addition, the expression of CCKBR is regarded as a symbol that has a close relationship with gastric canceration. IL-12β is a pro-inflammatory cytokine that is produced by various immune cells [86, 87]. The expression of IL-12β can be remarkably increased when the inflammatory response occurs in the layer of gastric tissues [88]. And studies have demonstrated that its regulatory gene IL12B is the target gene of NF-κB [89]. Importantly, the activation of NF-κB is critically responsible for the secretion of cytokines including IL-6, IL-1β, TNF-α and so on [90]. It has been reported that the extract of ZJW could significantly reduce the activity of NF-κB as well as some pro-inflammatory cytokines [91]. As is reported in the previous studies, ZJW was found to be an effective inhibitor for IL-1β, IL-6 and TNF-α in a concentration-dependent manner in LPS-induced RAW 264.7 macrophages [92]. It is also validated that ZJW could relieve the inflammation by downregulating the levels of TNF-α and IL-1β in rats, reducing the expression of the gene TNFA and IL1B in gastric mucosa [93]. And reducing cytokine IL-6 is another effective way for ZJW to attenuate the infiltration of immune cells and injury area in the stomach of rats [94]. It is evident that ZJW can upregulate the expression level of EGFR in rats with gastric ulcer induced by acetic acid, promoting the restoration of damaged gastric mucosa [95]. The down-regulation of ZJW for MCP-1 has been inferred as a result of anti-inflammatory effects of ZJW. Moreover, ZJW may exert its therapeutic effects through inhibiting the expression of IL-12β and CCKBR, cutting down the opportunity of further deterioration of gastritis. In summary, blocking these pro-inflammatory factors and raising relevant growth factors may be the pharmacological mechanism of ZJW acting on gastritis.

Besides, stimulation of growth and development signaling (EGFR signaling pathway, Gastrin in cell growth and proliferation and so on) leads to cell growth and proliferation. Excessive or inappropriate cell growth and proliferation can be one of the major causes of tumor development [96, 97]. It has been reported that excessive NF-κB activation was detected in human colorectal cancer tissues [98], and ZJW played a suppressive role in the expression of NF-κB [99]. Furthermore, an investigation concerning effects of ZJW on eight kinds of human cancer cell lines suggested that ZJW has significant anti-cancer activities due to up-regulation of pro-apoptosis proteins (caspase-3, caspase-9, Bax and Bak) and down-regulation of anti- apoptosis proteins (Bcl-2 and Bcl-xl) [100]. However, anti-cancer effects of ZJW in vivo and specific molecular mechanisms deserved to be deeply explored.

In addition, among the putative targets of ZJW, IL-6 is putative target of 2-Methoxy-4-vinylphenol and caffeine from *E. rutaecarpa*, IL-1β is putative target of alpha-humulene from *E. rutaecarpa*, TNF-α is putative target of hexanal, palmitic acid, rutaecarpine from *E. rutaecarpa* and quercetin from *R. coptidis*, EGFR is putative target of rutin from *E. rutaecarpa*, MCP-1 is putative target of quercetin from *R. coptidis*. These compounds are proposed to be the active constituents of ZJW in the treatment of gastritis.

However, there are also some limitations for the use of network pharmacology approach to predict active ingredients and potential mechanisms. (i) the active ingredients screened might be inconsistent with the ingredients actually absorbed in blood of the patients with gastritis; (ii) to distinguish inhibitory effects from activated effects of the targets could be a difficult problem; (iii) the predicted results might be impacted by possible biases to highly studied pathways/functions. Therefore, further experimental verification of the potential effective ingredients is demanded to validate theoretical predictions.

## Conclusion

TCM, one of the most important parts of complementary and alternative medicine, markedly contributes to the therapeutic action of gastrointestinal diseases. This study uses a scientific approach to holistically decipher that the pharmacological mechanisms of ZJW in the treatment of gastritis may be associated with its involvement into inflammation suppression and immune responses, growth and development pathways. Among these crucial biological functions, eight targets were identified as key active factors involved in the related pathways. However, to enhance the reliability of the results, further experimental experiments were demanded to validate these hypotheses.

## Additional files

Additional file 1: **Table S1.** Detailed information on eight existing protein-protein interaction databases. (XLSX 12 kb)

Additional file 2: **Table S2.** Ingredients of each herb contained in ZJW. (XLSX 1869 kb)

Additional file 3: **Table S3.** Known therapeutic targets for gastritis. (XLSX 14 kb)

Additional file 4: **Table S4.** Putative targets of each herb contained in ZJW and overlap between the two herbs. (XLSX 38 kb)

Additional file 5: **Table S5.** Interactions between putative targets of ZJW and known therapeutic target for gastritis. (XLSX 474 kb)

Additional file 6: **Table S6.** Major hubs in the network. (XLSX 14 kb)

Additional file 7: **Table S7.** Significant pathways. (XLSX 18 kb)

Additional file 8: **Table S8.** The whole null model according to Erdös-Rényi model. (XLSX 340 kb)

Additional file 9: **Table S9***.* The major hubs of null-model- network. (XLSX 8 kb)

## Abbreviations

AC: Adenylyl cyclase
AKT: Protein kinase B
Ca^2+^: Calcium ion
cAMP: Cyclic adenosine monophosphate
CASC: Chinese Academy of Sciences Chemistry
CCKBR: Cholecystokinin-B receptors
CCL2: CC-chemokine ligand 2
EGF: Epidermal growth factor
EGFR: Epidermal growth factor receptor
IL-12β: Interleukin 12 beta
IL-1β: Interleukin 1 beta
IL-6: Interleukin 6
MCP-1: Monocyte chemotactic protein-1
NF-κB: Nuclear factor kappa B
OMIM: Online Mendelian Inheritance in Man
PI3K: Phosphoinositide 3-kinase
PPI: Protein–protein interaction
TCMSP: Traditional Chinese Medicine Systems Pharmacology
TNF-α: Tumor necrosis factor alpha
ZJW: Zuojinwan
